# All You Need to Know and More about the Diagnosis and Management of Rare Mold Infections

**DOI:** 10.1128/mBio.02920-20

**Published:** 2021-02-23

**Authors:** Martin Hoenigl, Stuart M. Levitz, Audrey N. Schuetz, Sean X. Zhang, Oliver A. Cornely

**Affiliations:** a Section of Infectious Diseases and Tropical Medicine, Department of Internal Medicine, Medical University of Graz, Graz, Austria; b Division of Infectious Diseases and Global Public Health, Department of Medicine, University of California San Diego, San Diego, USA; c Clinical and Translation Fungal Research Working Group, University of California San Diego, San Diego, USA; d Department of Medicine, University of Massachusetts Medical School, Worcester, Massachusetts, USA; e Department of Laboratory Medicine and Pathology, Mayo Clinic, Rochester, Minnesota, USA; f Microbiology Laboratory, Johns Hopkins Hospital, Johns Hopkins University, Baltimore, Maryland, USA; g Department of Pathology, School of Medicine, Johns Hopkins University, Baltimore, Maryland, USA; h University of Cologne, Faculty of Medicine, Cologne, Germany; i University Hospital Cologne, Department I of Internal Medicine, Excellence Center for Medical Mycology (ECMM), Cologne, Germany; j University Hospital Cologne, Translational Research, Cologne Excellence Cluster on Cellular Stress Responses in Aging-Associated Diseases (CECAD), Cologne, Germany; k University Hospital Cologne, Clinical Trials Centre Cologne (ZKS Köln), Cologne, Germany; l German Centre for Infection Research (DZIF), Partner Site Bonn-Cologne, Cologne, Germany

**Keywords:** *Fusarium*, *Lomentospora*, *Paecilomyces*, *Penicillium*, *Phaeohyphomycosis*, *Rasamsonia*, *Scedosporium*, *Scopulariopsis*, basidiomycetes, diagnosis, molds, treatment

## Abstract

Invasive mold infections caused by molds other than *Aspergillus* spp. or Mucorales are emerging. The reported prevalences of infection due to these rare fungal pathogens vary among geographic regions, driven by differences in climatic conditions, susceptible hosts, and diagnostic capabilities. These rare molds—*Fusarium*, *Lomentospora*, and *Scedosporium* species and others—are difficult to detect and often show intrinsic antifungal resistance. Now, international societies of medical mycology and microbiology have joined forces and created the “Global guideline for the diagnosis and management of rare mould infections: an initiative of the European Confederation of Medical Mycology in cooperation with the International Society for Human and Animal Mycology and the American Society for Microbiology” (published in Lancet Infect Dis, https://doi.org/10.1016/S1473-3099(20)30784-2), with the goal of improving the diagnosis, treatment, prevention, and survival of persons with rare mold infections. The guideline provides cutting-edge guidance for the correct utilization and application of established and new diagnostic and therapeutic options.

## COMMENTARY

The fungal bloom at the end of the Cretaceous Period has favored the selection of endothermic mammals over ectothermic reptiles, because the warm body temperatures of mammals protected them from fungal diseases (1). Since then, the world has become a warmer place, and an increasing number of fungal species are adapting to high temperatures and are emerging as important human pathogens (2). Due to advances of modern medicine, new groups of patients at risk of developing invasive fungal diseases (IFD), and particularly invasive mold disease, have been identified. These patients include those with severe acute respiratory syndrome coronavirus 2 or influenza virus A or B, requiring intensive care (3, 4), and those receiving biological therapies (such as tumor necrosis factor alpha inhibitors) and new small-molecule kinase inhibitors (such as ibrutinib and idelalisib) (5). Mold-active antifungal prophylaxis in those at highest risk for invasive aspergillosis has proven effective in preventing invasive aspergillosis and, to a lesser extent, also mucormycosis (6, 7). However, the selective pressure of antifungal prophylaxis may also contribute to the emergence of less common invasive mold infections, caused by molds that are often intrinsically resistant to some classes of antifungals, including *Fusarium*, *Lomentospora*, and *Scedosporium* species, as well as even less common emerging molds, such as *Rasamsonia*, *Schizophyllum*, *Scopulariopsis*, *Purpureocillium*, and *Paecilomyces* spp., which have been described as opportunistic pathogens in patients with a variety of underlying diseases (8–10). The intrinsic resistance of the fungal pathogens to many of the available antifungals limits successful therapeutic options (11, 12). The reported prevalences of infection due to these rare fungal pathogens vary widely among geographic regions, driven by differences in climatic conditions, susceptible hosts, and diagnostic capabilities (10–14). The increasing availability of new nucleic acid amplification assays, blood culture detection systems, and lateral flow devices, as well as matrix-assisted laser desorption ionization–time of flight (MALDI-TOF) mass spectrometry for the detection of medically important fungi and the laboratory diagnosis of invasive mycoses, warrants guidance in their use in clinical practice. Also, therapeutic options differ across global regions, and guidance needs to reflect this to optimize patient management.

Now, international societies of medical mycology and microbiology have joined forces with the goal of improving the diagnosis, treatment, prevention, and survival of persons with rare mold infections. For the in-press “Global Guideline for the Diagnosis and Management of Rare Mold Infections: an Initiative of the European Confederation of Medical Mycology (ECMM) in Cooperation with the International Society for Human and Animal Mycology (ISHAM) and the American Society for Microbiology (ASM)” (15), medical professionals from around the world, representing all United Nations regions and all medical disciplines involved in the management of invasive mold diseases, contributed their expertise and analyzed published evidence to develop global guidance for the diagnosis and management of rare mold infections. During the 6-week public review phase, over 350 comments and suggestions were submitted and subsequently incorporated into the guideline. Specifically, the 3 reviewers appointed by the ASM provided outstanding detailed reviews, which further improved the diagnostic, laboratory, and treatment aspects of the guideline significantly. The guideline takes into account that available diagnostic and therapeutic options differ across global regions and gives out recommendations stratified for high- and low-resource countries. Evidence-based diagnostic strategies and approaches, both primary and ancillary in nature, are clearly delineated in this document. Advantages and disadvantages of various diagnostic methods are outlined and will prove helpful for laboratorians and clinicians alike. The inclusion of excellent photomicrographs of microscopic morphologies obtained from colonial growth, as well as typical histopathologic appearances in tissue, provides a wealth of information on these rare pathogens for anatomic pathologists and medical microbiologists. The present recommendations comprise the second guidance document of the One World-One Guideline initiative (after the mucormycosis guideline, which was published in 2019) (16, 17) to incorporate regional differences in epidemiology and management, and the guidance document was reviewed and endorsed by 54 scientific societies, including national societies from 38 countries and several international societies.

While previous guidelines in this area (i) were limited to individual rare mold pathogens (18–20) and focused on specific groups of patients, such as those with hematological malignancies (20), or (ii) were missing altogether for infections caused by many of the very rare, but emerging, pathogenic molds, this new comprehensive guideline overcomes these problems by leveraging online resources, including shared folders, video- and teleconferences, YouTube tutorials (e.g., https://www.ecmm.info/guidelines/), and training materials, which have become essential because of the fact that face-to-face meetings in the context of worldwide contributions are not feasible (which was true even before the coronavirus disease 2019 pandemic) and to ensure short timelines to guideline completion. In fact, the short timeline to completion is a strength of the current guideline on rare molds, which includes evidence that came out during recent months of 2020, such as the superiority of lomentosporiosis treatment with voriconazole and terbinafine combination versus monotherapy (11).

Utilization of new online tools to facilitate worldwide exchange and knowledge sharing, including educational videos, are now indispensable in our increasingly global world. By using these online resources, ambitious goals like worldwide guidelines suddenly become feasible, as communication and decision-making are transparent, quick, and not restricted to planned, comparatively expensive in-person meetings, where a major challenge is to bring together experts from all regions around the globe to discuss matters.

In conclusion, in the context of a growing population of immunocompromised patients at risk of opportunistic infections, the prevalence of invasive fungal disease caused by emerging and often drug-resistant rare molds is on the rise. While new diagnostic and therapeutic options are now available to tackle IFD, cutting-edge guidance for their correct utilization and application in a range of clinical settings is provided within this guideline document, focusing on infections caused by *Fusarium* spp., *Lomentospora* spp., *Scedosporium* spp., dematiaceous molds causing phaeohyphomycosis, *Rasamsonia* spp., *Scopulariopsis* spp., *Penicillium* spp., non-*marneffei Talaromyces* spp., *Paecilomyces* spp., *Purpureocillium* spp., and *Schizophyllum* spp., as well as other basidiomycetes.
